# Spontaneous nanoemulsification for solubility enhancement of BCS class II and IV molecules, quercetin as a model drug

**DOI:** 10.1016/j.mex.2025.103298

**Published:** 2025-04-01

**Authors:** Marwan Motuq Alsufyani, Waleed Mohammad Alqarni, Yousef Alzahrani, Alaa Khalid Balbed, Musab Musleh Alkathyri, Mohammed Akhlaquer Rahman

**Affiliations:** Department of Pharmaceutics and Industrial Pharmacy, College of Pharmacy, Taif University, Taif 21974, Saudi Arabia

**Keywords:** Aqueous titration, Ternary-phase diagram, Nanoemulsion, Solubility, Quercetin, Spontaneous nanoemulsification for solubility enhancement of BCS class II and IV molecules

## Abstract

Spontaneous emulsification represents a practicable and efficient method for the formulation of nanoemulsion utilized in drug delivery systems. This method provides numerous advantages, such as increased energy efficiency optimization, the possibility of scaling up for industrial use, enhanced drug loading capacity, and safeguarding sensitive compounds designated for encapsulation. Nanoemulsion can be produced simply by combining water, oil, surfactant, and co-surfactant in specific ratios. The sequence in which these components are mixed is generally regarded as non-critical, as nanoemulsions form spontaneously. However, despite the spontaneous nature of nanoemulsification, the forces driving this process are minimal, and the time required for these systems to achieve equilibrium can be considerable. The titration method employed for developing the phase diagram, along with the selection of nanoemulsions from the constructed phase diagram, is crucial for researchers. The objective of this study is to understand the feasibility of this method to prepare nanoemulsion using quercetin as model drug. Overall,

• This method resulted in development of efficient screening technique for nanoemulsion.
• Validation was achieved by measuring droplet size and drug release confirmed the usefulness of the method.
• This approach presents a cost-effective method applicable in drug design to enhance its solubility and thus bioavailability.

## Graphical abstract

**Figure:**
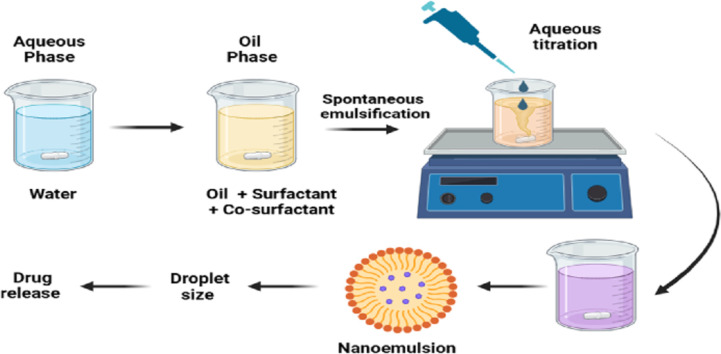

Specifications table

**Table:** 

Subject area:	Pharmacology, Toxicology and Pharmaceutical Science
More specific subject area:	Nanomedicine
Name of your method:	Spontaneous nanoemulsification for solubility enhancement of BCS class II and IV molecules
Name and reference of original method:	Not Applicable
Resource availability:	All resources required included in the present article

## Background

Recent estimates indicate that as much as 40 % of new chemical entities (NCEs) discovered by the pharmaceutical sector exhibit poor aqueous solubility or lipophilicity. This situation leads to various challenges, including low oral bioavailability, significant variability among individuals, and issues with dose proportionality [1,2]. For these compounds, the rate of absorption in the gastrointestinal tract (GIT) is largely dissolution-dependent [3]. The need to address poor solubility of drug is becoming increasingly critical as pharmaceutical companies rely more profoundly on NCEs for revenue generation. Solubility challenges not only affect emerging drugs but also impact the delivery of many existing medications. Generic drug manufacturers are required to implement cost-effective delivery methods to maintain their competitiveness, particularly as an increasing number of low-solubility drugs approach the expiration of their patent protections in this price-sensitive market. In response to these challenges, a variety of strategies have been utilized to improve the solubility of poorly water-soluble drugs, employing diverse techniques for the development of an efficient drug delivery system.

Nanomedicine has gained prominence in recent decades as a significant area of research, primarily due to its benefits compared to traditional therapeutic approaches [4,5]. Among the various drug delivery systems in nanomedicine, nanoemulsions (NEs) stand out as crucial vehicles, facilitating the efficient delivery of lipophilic drugs encapsulated within the oily core of the droplets [6]. NEs can be produced through various techniques, which are generally categorized into high energy and low energy methods [7]. The most widely utilized high-energy techniques include high-pressure homogenization, micro-fluidization, and ultrasonication [8]. In contrast, low energy methods, often referred to as transitional nanoemulsification techniques, primarily encompass phase inversion temperature methods and spontaneous emulsification [9]. The benefits of low energy methods compared to high energy techniques are evident in the increased energy yields, which are advantageous for scaling up industrial processes. Additionally, low energy processes are inherently gentle, thereby minimizing the risk of denaturation or damage to delicate molecules [10].

**Fig. 1:**
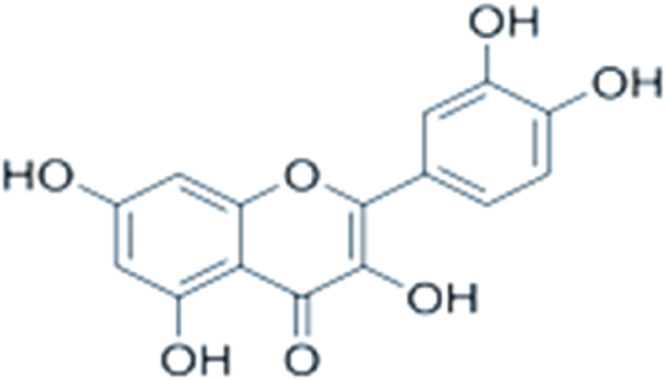
Chemical structure of quercetin. solubility: 2.15 mg/L at 25 °C to 665 mg/L at 140 °C.

Quercetin (Fig. 1), a naturally occurring compound belonging to BCS class II and IV category [11,12], known for its diverse pharmacological properties, highlights the difficulties encountered with drugs that have low aqueous solubility. Beyond its potential in cancer treatment, quercetin demonstrates a variety of advantageous biological effects, such as antioxidant activity, the ability to scavenge free radicals, and antiviral properties [13]. The administration of quercetin encounters several obstacles, such as inadequate oral absorption and bioavailability, the potential for embolization from intravenous administration due to drug precipitation, local tissue toxicity, and its chemically unstable nature, especially in alkaline aqueous environments [14]. Although acidic conditions offer some degree of protection against degradation, quercetin is significantly metabolized in the GIT and liver, resulting in metabolites that possess some residual biological activity [15,16]. These elements collectively lead to the remarkably low oral bioavailability of unaltered quercetin in humans [17].

The current research aimed to formulate and assess stable nanoemulsion formulations of quercetin, incorporating oil, surfactant, and co-surfactant through the spontaneous emulsification method. This approach is becoming increasingly significant due to its simplicity in execution at the laboratory level and the absence of the need for high shear equipment, which may induce thermodynamic instability in the system.

## Method details

This method is a comprehensive analysis of the spontaneous emulsification method that generates nanoemulsions intended for oral administration. Nanoemulsions are easy to prepare by this method and require no significant energy contribution during preparation and thus scale up technology is easy. The methodology elucidates the principles behind the calculation and development of pseudo ternary phase diagrams. Most notably, it facilitates the selection of formulations from these phase diagrams to circumvent metastable formulations, ensuring minimal surfactant concentration was achieved in the shortest time frame. Different steps are involved in this method that includes.

### Solubility assessment of quercetin in different excipients

Solubility study was performed by UV spectral analysis in order to recognize the suitable excipients for the preparation of nanoemulsion. The equilibrium solubility of quercetin in different oils, surfactant and co-surfactant was determined by addition of an excess amount of drug in 2 mL of excipients in 5 mL glass stoppered vials. The glass vials were then placed in an isothermal shaker (Nirmal International, India) maintained at 25 °C for 72 h. The samples after equilibrium were removed from shaker and centrifuged (REMI, Ultracentrifuge) at 3000 rpm for 15 min. The supernatant was collected and clarified through a 0.45 µm membrane filter. The amount of drug (mg/mL) was determined in each excipients using UV spectroscopy (UV-1700 spectrophotometer, Shimadzu, Japan) at 369 nm.

### Construction of ternary phase diagram and fabrication of nanoemulsion

**Fig. 2:**
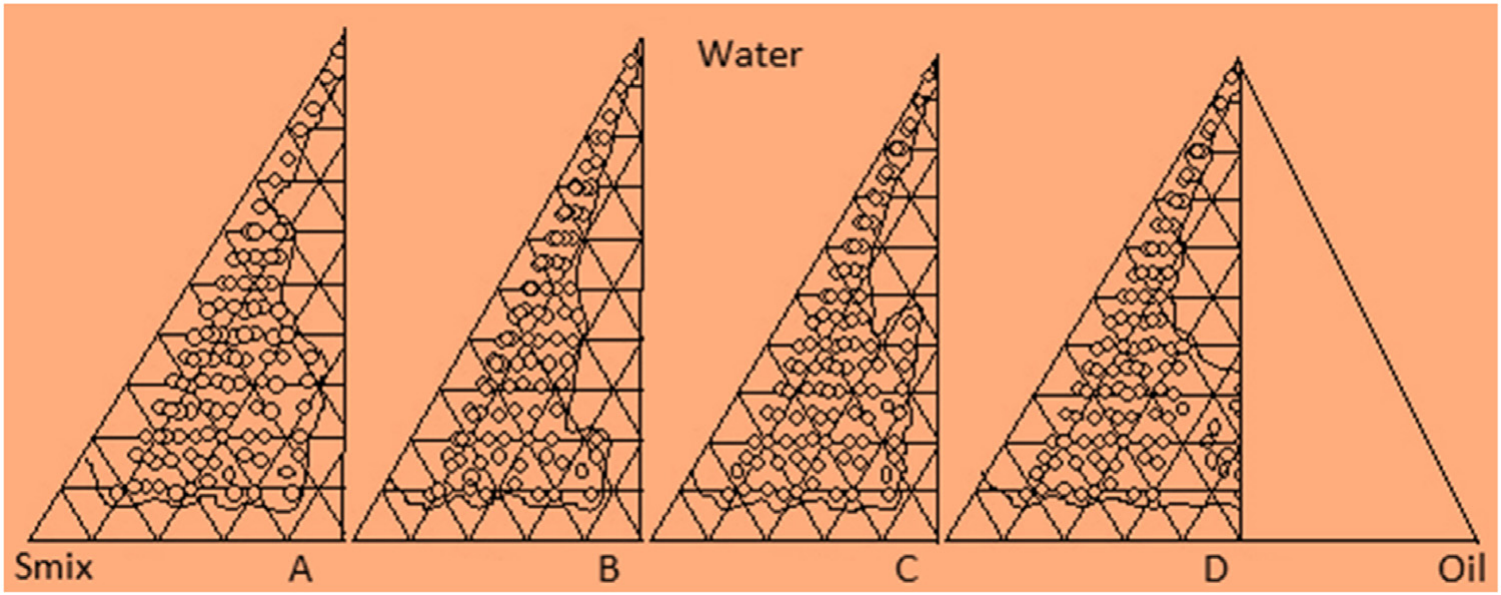
Pseudo ternary phase diagram consisting of oil, S_mix_ and water. Each corner of the diagram represents 100 % of that particular component. Fig. 2 (A, B, C and D) represent phase diagram with S_mix_ ratio 1:1, 1:2, 2:1, and 3:1, respectively.

Surfactant and co-surfactant (S_mix_) were combined in various volume ratios (1:1, 1:2, 2:1, 3:1). These specific S_mix_ ratios were selected to progressively increase the concentration of the co-surfactant relative to the surfactant, as well as to increase the concentration of the surfactant in relation to the co-surfactant, facilitating a comprehensive analysis of the phase diagrams involved in nanoemulsion formation. For each phase diagram, oil and a specific S_mix_ ratio were thoroughly mixed in various volume ratios ranging from 1:9 to 9:1 in different glass vials. A total of sixteen distinct combinations of oil and S_mix_ were prepared, including ratios of 1:9, 1:8, 1:7, 1:6, 1:5, 2:8, 1:3.5, 1:3, 3:7, 1:2, 4:6, 5:5, 6:4, 7:3, 8:2, and 9:1, ensuring that the maximum ratios were represented to accurately define the phase boundaries illustrated in the phase diagrams. Pseudo ternary phase diagrams were constructed utilizing the aqueous titration method (Fig. 2). From each constructed phase diagram, various formulations were chosen from the nanoemulsion region to facilitate the straightforward incorporation of a single drug dose into the oil phase. Based on the pseudo ternary phase diagrams that exhibited the largest nanoemulsion area, several nanoemulsions with distinct formulations were identified. The selection encompassed nearly the entire spectrum of nanoemulsion presence within the phase diagrams, focusing on different oil compositions that utilized minimal surfactant and maximized water concentration, thereby demonstrating the existence of nanoemulsions (Table 1). The oil phase was taken, followed by the addition of the necessary quantity of surfactant and co-surfactant. Water was then introduced gradually, drop by drop, until a clear and transparent liquid was achieved. The resulting nanoemulsion was securely sealed and stored at room temperature.

**Table 1:** Thermodynamic and dispersibility test of selected formulations from S_mix_ ratio 1:1, 1:2, 2:1, 3:1.

S_mix_ Ratio (S:CoS)	Percentage v/v of different components in formulation			Thermodynamic stability test			Inference	Dispersibility test		Inference
	Oil	S_mix_	Water	H/C	Cent	Freez		DW	PB	
**1:1 (**Fig. 2**A)**	10	35	55	–	√	X	Failed	–	–	–
	10	40	50	√	√	√	Passed	Grade A	Grade A	Passed *
	15	40	45	√	√	√	Passed	Grade A	Grade A	Passed *
	15	45	40	√	√	√	Passed	Grade A	Grade C	Failed
	20	40	40	√	√	√	Passed	Grade A	Grade B	Passed
	20	45	35	√	√	√	Passed	Grade B	Grade C	Failed
	25	42	33	√	√	√	Passed	Grade A	Grade C	Failed
	25	45	30	√	√	√	Passed	Grade B	Grade C	Failed
**1:2 (**Fig. 2**B)**	10	30	60	x	√	√	Failed	–	–	–
	10	35	55	√	√	√	Passed	Grade A	Grade A	Passed *
	15	38	47	√	√	√	Passed	Grade A	Grade A	Passed *
	15	40	45	√	√	√	Passed	Grade A	Grade B	Passed
	20	40	40	√	√	√	Passed	Grade A	Grade C	Failed
	20	45	35	√	√	√	Passed	Grade B	Grade B	Failed
	25	40	35	–	x	√	Failed	–	–	–
	25	45	30	–	x	√	Failed	–	–	–
**2:1 (**Fig. 2**C)**	10	32	58	√	√	√	Passed	Grade A	Grade B	Passed
	10	35	55	√	√	√	Passed	Grade A	Grade A	Passed *
	15	40	45	–	x	–	Failed	–	–	–
	15	42	43	√	√	√	Passed	Grade A	Grade C	Failed
	20	40	40	√	√	√	Passed	Grade A	Grade B	Passed
	20	45	35	√	√	√	Passed	Grade A	Grade C	Failed
	25	40	35	√	√	√	Passed	Grade A	Grade C	Failed
	25	42	33	√	√	√	Passed	Grade B	Grade C	Failed
**3:1 (**Fig. 2**D)**	10	38	52	–	x	–	Failed	–	–	–
	10	40	50	–	x	–	Failed	–	–	–
	15	45	40	–	x	–	Failed	–	–	–
	15	48	38	√	√	√	Passed	Grade A	Grade C	Failed
	20	40	40	–	√	X	Passed	Grade A	Grade C	Failed
	20	45	35	√	√	√	Passed	Grade A	Grade B	Passed
	25	40	35	–	x	–	Failed	–	–	–
	25	45	30	√	√	√	Passed	Grade A	Grade C	Failed

⁎ Passed nanoemulsion formulation after thermodynamic and dispersibility test in Grade A designated as NE1 (Oil: 10 % v/v, S_mix_: 40 % v/v, Water: 50 % v/v), NE2 (Oil: 15 % v/v, S_mix_: 40 % v/v, Water: 45 % v/v), NE3 (Oil: 10 % v/v, S_mix_: 35 % v/v, Water: 55 % v/v), NE4 (Oil: 15 % v/v, S_mix_: 38 % v/v, Water: 47 % v/v), and NE5 (Oil: 10 % v/v, S_mix_: 35 % v/v, Water: 55 % v/v).

### Thermodynamic stability test

Heating cooling cycle: Six heating and cooling cycles of storage were conducted between a refrigerator temperature of 4 °C and 45 °C, with each temperature maintained for a minimum of 48 h. Formulations that remained stable at these temperatures were then subjected to a centrifugation test. Centrifugation: The formulations that passed this test were centrifuged at 3500 rpm for 30 min. Formulations that did not exhibit any phase separation were selected for the freeze-thaw stress test. Freeze thaw cycle: This involved three freeze-thaw cycles between −21 °C and +25 °C. Those formulations that successfully passed these thermodynamic stress tests (as detailed in Table 1) were subsequently evaluated for their dispersibility to assess the efficiency of self-emulsification.

### Dispersibility test

Self-emulsification efficiency of oral nanoemulsion was evaluated utilizing a standard dissolution apparatus (USP apparatus II). 1 mL of each formulation was introduced into 500 mL of distilled water (DW) and phosphate buffer (PB) at pH 7.4, maintained at a temperature of 37 ± 0.5 °C. A standard stainless steel dissolution paddle, rotating at 50 rpm, facilitated gentle agitation. The *in vitro* performance of the formulation was visually evaluated according to a specified grading system.

Grade A: A nanoemulsion that forms quickly (within 1 min) and displays a clear or bluish hue;

Grade B: A rapidly forming emulsion that is somewhat less clear, presenting a bluish-white color;

Grade C: A fine milky emulsion that develops within 2 min;

Grade D: A dull, grayish-white emulsion with a slightly oily look that emulsifies slowly (taking longer than 2 min);

Grade E: A formulation characterized by either inadequate or minimal emulsification, with large oil droplets visible on the surface.

Formulations that successfully met the criteria for thermodynamic stability and dispersibility tests, achieving Grade A or B, were selected for further investigation (refer to Table 1). From each group, one formulation was chosen at each oil concentration level (10 %, 15 %, 20 %, and 25 %), characterized by the lowest S_mix_ concentration, regardless of the S_mix_ ratio employed, provided that they passed the dispersibility test with a Grade A or B rating.

### Droplet size analysis

The droplet size of the nanoemulsion was assessed using photon correlation spectroscopy, which evaluates the variations in light scattering resulting from the Brownian motion of the particles. A formulation of 0.1 mL was diluted in 50 mL of water in a volumetric flask and mixed gently by inverting the flask. Measurements were conducted using a Zetasizer 1000 HS (Malvern Instruments, UK). Light scattering was observed at a temperature of 25 °C and at an angle of 90°

### In-vitro drug release

An *in vitro* release test was conducted using 500 mL of dissolution media, specifically a phosphate buffer at pH 7.4, following the USP XXIV method. The test utilized dissolution apparatus (USP II), operating at 50 rpm and a temperature of 37 ± 0.5 °C. A volume of 1 mL of the nanoemulsion formulation was introduced into a treated dialysis bag with a molecular weight cut-off of 12,000 g/mole (Sigma, USA). At predetermined time intervals (0, 1, 2, 4, 6, 12, 24, and 48 h), 1 mL sample was taken, and an equal volume of phosphate buffer was added to maintain the sink condition. The drug content in the sample was assessed using UV spectroscopy at a wavelength of 369 nm. The drug release profiles from the nanoemulsion formulations were compared to those of a pure drug suspension.

## Method validation

**Fig. 3:**
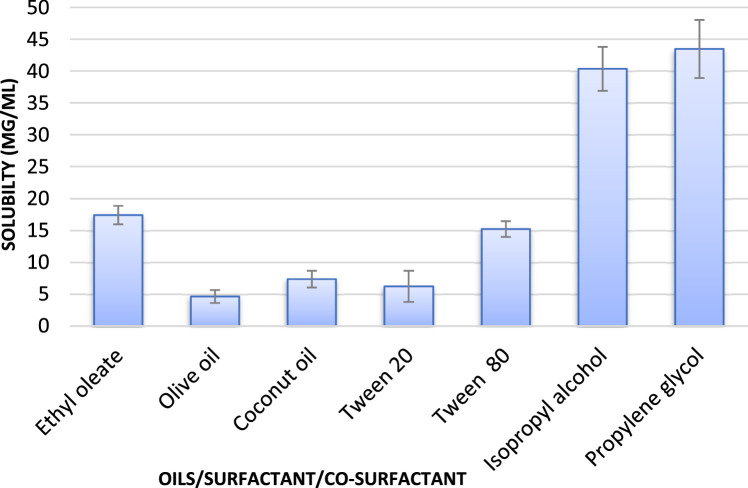
Solubility of quercetin in various excipients. Ethyl oleate: 17.44 ± 1.45 mg/mL; Olive oil: 4.64 ± 1.02 mg/mL; Coconut oil: 7.37 ± 1.37 mg/mL; Tween 20: 6.24 ± 2.45 mg/mL; Tween 80; 15.27 ± 1.25 mg/mL; Isopropyl alcohol; 40.34 ± 3.45 mg/mL; Propylene glycol: 43.46 ± 4.56 mg/mL.

Before starting the phase diagram, one must have to select suitable excipients in which the drug shows maximum solubility to be in the desired solubility range which is essential for the formulation of nanoemulsion drug delivery system. On the basis of solubility study it was concluded that the solubility of quercetin in ethyl oleate was found to be favorable (Fig. 3) for the nanoemulsion preparation and was selected as oil phase. Tween 80 and propylene glycol was also selected on the basis of solubility. The selected oils come under the generally regarded as safe category.

The size of the droplets in a nanoemulsion serves as a key criterion for confirming that the nanoemulsions produced by this method fall within the nanometer range (100 nm). The average droplet size for formulations NE1, NE2, NE3, NE4, and NE5 were recorded as 58.8 ± 1.17 nm, 62.6 ± 2.08 nm, 59.8 ± 1.63 nm, 64.4 ± 1.15 nm, and 67.2 ± 1.92 nm, respectively. The variations in droplet size among these formulations were not statistically significant (*p* > 0.05). The minor differences observed in the mean size of the oil globules may be attributed to an inadequate concentration of the S_mix_ and the specific composition of the S_mix_ ratio.

In order to validate that the nanoemulsion produced by this method enhanced the solubility of quercetin, a release study was conducted. The release profile was assessed in simulated intestinal fluid at a pH of 7.4. The rate of drug release from all nanoemulsion formulations were significantly higher (p<0.05) compared to the plain drug suspension. The formulation NE1 exhibited the highest release, achieving 98.23 ± 9.37 %, in comparison to the other formulations, NE2 (96.23 ± 7.48 %), NE3 (97.23 ± 8.35 %), NE4 (95.23 ± 7.45 %), and NE5 (96.45 ± 8.42 %) at 48 h. In contrast, the plain drug suspension released only 36.23 ± 3.36 % of the drug over the same 48 h period, attributed to its low aqueous solubility. In conclusion, the method adopted may offer viable alternatives to high-energy methods and could serve as a foundational approach for designing intricate vehicles for encapsulating hydrophilic therapeutic agents.

## Limitations

Construction of phase diagrams is time consuming process, especially when the goal is to accurately delineate a phase-boundary, as the time taken for the system to equilibrate can be significantly increased as the phase boundary is approached. Care must be taken to ensure that observations are not made on metastable systems, although the free energy required to form an emulsion is very low, the formation is thermodynamically spontaneous. It is evident that time constraints impose a physical limit on how long systems may be allowed to equilibrate, and as a result, it might be challenging to ensure the removal of metastable states in practice.

## CRediT authorship contribution statement

**Marwan Motuq Alsufyani:** Conceptualization, Methodology, Writing – review & editing. **Waleed Mohammad Alqarni:** Conceptualization, Methodology, Writing – review & editing. **Yousef Alzahrani:** Conceptualization, Methodology, Writing – review & editing. **Alaa Khalid Balbed:** Conceptualization, Methodology, Writing – review & editing. **Musab Musleh Alkathyri:** Conceptualization, Methodology, Writing – review & editing. **Mohammed Akhlaquer Rahman:** Conceptualization, Methodology, Supervision, Writing – original draft, Writing – review & editing.

## Data Availability

Data will be made available on request.

## References

[1] Aungst B.J. (1993). Novel formulation strategies for improving oral bioavailability of drugs with poor membrane permeation or pre-systemic metabolism. J. Pharm. Sci..

[2] Robinson J.R. (1996). Introduction: semi-solid formulations for oral drug delivery. B. T. Gattefosse.

[3] Amidon G.L., Lennernas H., Shah V.P., Crison J.R. (1995). A theoretical basis for a biopharmaceutic drug classification: the correlation of *in vitro* drug product dissolution and *in vivo* bioavailability. Pharm. Res..

[4] Mirza A.Z., Siddiqui F.A. (2014). Nanomedicine and drug delivery: a mini review. Int. Nano Lett..

[5] Rahman M.A. (2025). Exploration of nanomedicine-based dry powder inhalation formulation co-loaded with erlotinib and curcumin for treatment of non-small cell lung cancer. Nano LIFE.

[6] Mou D., Chen H., Du D., Mao C., Wan J., Xu H., Yang X. (2008). Hydrogel-thickened nanoemulsion system for topical delivery of lipophilic drugs. Int. J. Pharm..

[7] Akram S., Wang X., Vandamme T.F., Collot M., Rehman A.U., Messaddeq N., Mély Y., Anton N. (2019). Toward the formulation of stable micro and nano-double emulsions through a silica coating on internal water droplets. Langmuir.

[8] Anton N., Benoit J.P., Saulnier P. (2008). Design and production of nanoparticles formulated from nanoemulsion templates-a review. J. Control. Release.

[9] Anton N., Akram S., Vandamme T.F. (2018). Transitional nanoemulsification methods. Nanoemulsions.

[10] Gulotta A., Saberi A.H., Nicoli M.C., McClements D.J. (2014). Nanoemulsion-based delivery systems for polyunsaturated (ω-3) oils: formation using a spontaneous emulsification method. J. Agric. Food Chem..

[11] Salehi B., Machin L., Monzote L., Sharifi-Rad J., Ezzat S.M., Salem M.A., Merghany R.M., El Mahdy N.M., Kılıç C.S., Sytar O., Sharifi-Rad M., Sharopov F., Martins N., Martorell M., Cho W.C. (2020). Therapeutic potential of quercetin: new insights and perspectives for human health. ACS Omega.

[12] Manzoor M.F., Hussain A., Sameen A., Sahar A., Khan S., Siddique R., Aadil R.M., Xu B. (2021). Novel extraction, rapid assessment and bioavailability improvement of quercetin: a review. Ultrason. Sonochem..

[13] Formica J.V., Regelson W. (1995). Review of the biology of quercetin and related bioflavonoids. Food Chem. Toxicol..

[14] Rahman M.A., Mittal V., Wahab S., Alsayari A., Muhsinah A.B., Almaghaslah D. (2022). Intravenous nanocarrier for improved efficacy of quercetin and curcumin against breast cancer cells: development and comparison of single and dual drug loaded formulations using hemolysis, cytotoxicity and cellular uptake studies. Membranes.

[15] Manach C., Morand C., Crespy V., Demigne C., Taxier O., Regerat F., Remesy C. (1998). Quercetin is recovered in human plasma as conjugated derivatives which retain antioxidant properties. FEBS Lett..

[16] Kim M., Sedykh A., S.K Chakravarti, Saiakhov R.D., Zhau H. (2014). Critical evaluation of human oral bioavailability for pharmaceutical drugs by using various cheminformatics approaches. Pharm. Res..

[17] Kawakami K., Yoshikawa T., Moroto Y., Kanaoka E., Takahashi K., Nishihara Y., Masuda K. (2002). Microemulsion formulation for enhanced absorption of poorly soluble drugs II. *In vivo* study. J. Control. Release.

